# Recurrent Herpes Zoster Ophthalmicus Preceded by Anabolic Steroids and High-Dose L-Arginine

**DOI:** 10.1155/2020/8861892

**Published:** 2020-12-28

**Authors:** Stephen A. LoBue, Adam Goldman, Richard A. Giovane, Stacy M. Carlson, Michael Bivona, Sinan Albear, Thomas D. LoBue

**Affiliations:** ^1^Department of Ophthalmology, SUNY Downstate, Brooklyn, NY 11203, USA; ^2^LoBue Laser & Eye Medical Centers, Murrieta, CA 92562, USA; ^3^LV Stabler Hospital, Greenville, AL 36037, USA; ^4^Montefiore Medical Center, Bronx, NY 10467, USA

## Abstract

**Purpose:**

To report a case of a 34-year-old male with recurrent herpes zoster ophthalmicus (HZO) preceded by a 6-week cycle of anabolic steroids and high-dose amino acid supplementation. *Case Presentation*. A 34-year-old man presented to our institution for left eye pain for one week associated with a vesicular rash in the V1 dermatome, respecting the midline. The patient had no significant past medical or past ocular history, including systemic immunosuppressive agents or HIV. However, prior to the onset of his symptoms the patient had completed a 6-week course of anabolic steroids including trenbolone, deca-durabolin, and testosterone as well as high-dose arginine supplementation averaging more than 40 grams a day. The best-corrected vision was 20/25 OS with slit-lamp examination remarkable for punctate staining and pseudodendrites at 6 o'clock, outside the visual axis. The patient was treated with oral acyclovir 800 mg five times a day for seven days along with prednisolone QID and moxifloxacin QID which was tapered over a month. Four months after resolution, the patient developed a recurrent HZO keratitis preceded by another cycle of anabolic steroids and amino acid supplementation.

**Conclusion:**

In vitro L-arginine supplementation has been associated with the proliferation and virulence of a variety of herpes viruses. Anabolic steroids have also been demonstrated by various studies to negatively affect cell-mediated immunity necessary to prevent viral infection. Thus, it is possible that anabolic steroids in conjunction with increased L-arginine intake may have precipitated a recurrent HZO in a previously healthy, immunocompetent individual.

## 1. Introduction

Anabolic-androgenic steroids (AAS), or performance-enhancing drugs, have been used by elite professional athletes and general fitness enthusiasts in order to increase strength and muscle growth. However, the prevalence of anabolic abuse within the USA is not known. Several factors may contribute to the underestimation of anabolic steroid users including insufficient drug tests, on and off cycling of AAS, and taboo or illegal connotation associated with AAS.

AAS comprises a group of synthetic and natural hormones which are structurally and functionally similar to testosterone. Despite the benefits associated with AAS, there are numerous well-known side effects including psychosis, organ damage (e.g., heart, liver, and kidney), hair loss, severe acne, increased risk of tendinitis or tendon rupture, and liver tumors [1]. Also, certain types of AAS have been documented to have immunosuppressive features, impairing B and T cell functions necessary for viral infections [2, 3].

Herpes zoster or shingles is the product of the reactivation of latent varicella zoster virus (VZV). Reactivation of the virus is common in the elderly and immunocompromised, with the rate of incidence increasing in each decade of life and peaking around 80 years old. Approximately 30% of all individuals will develop at least one shingles outbreak in their lifetime [4]. VZV can also affect the eye during the involvement of the ophthalmic division of the fifth cranial nerve (V1), known as herpes zoster ophthalmicus (HZO).

Nevertheless, we noticed an unusual presentation of recurrent HZO in a 34-year-old male, preceded by a 6-week cycle of anabolic steroids and high-dose amino acid supplementation prior to each episode.

## 2. Case Presentation

A 34-year-old man with no past medical history presented to our clinic complaining of left eye pain and pressure for one week. He was referred by his primary care doctor for suspected HZO, who had started the patient on acyclovir 800 mg 5 times a day prior to his visit to our clinic.

The patient denied any relevant past medical history including diabetes and HIV. He denied smoking, alcohol, or illicit drug use. The patient also denied taking any immunosuppressant agents including prednisone. However, the patient was an active weightlifter who reported having completed a 6-week course of AAS two weeks prior to the onset of his symptoms. His regimen consisted of trenbolone 200 mg, deca-durabolin 200 mg, and testosterone 500 mg a week (Table 1) along with a diet high in protein and supplemental amino acids including arginine. A diet recall averaged above 40 grams of L-arginine a day (Table 2).

On external exam, vesicles were noted over the left eyebrow and upper lid. Anterior segment exam was notable for decreased corneal sensation OS. Slit-lamp examination was remarkable for punctate staining and pseudodendrites at 6 o'clock, outside the visual axis OS. No anterior segment inflammation was noted with intraocular pressure within normal limits. The fundus exam was unremarkable for both eyes.

The patient was continued on oral acyclovir as well as moxifloxacin 0.5% QID and prednisone 1% QID OS which was tapered over a month. The patient was strongly encouraged to stop his AAS regiment as well as high-dose arginine diet/supplementation. HZO keratitis resolved over the course of a month.

Four months later, the patient presented to the clinic with new eye pain OS after completing another cycle of the above AAS and high-arginine diet. Uncorrected visual acuity was 20/40. Slit-lamp examination revealed deep stromal vessels with a 2 × 2 mm infiltrate just inferior to the visual axis at 5 o'clock. Intraocular pressure and fundus exam were unremarkable. The patient was started on acyclovir 800 mg 5 times a day for the next seven days with prednisolone 1% QID. The patient was counseled on stopping AAS as well as the need for a lifelong prophylaxis dose of acyclovir. However, after interval improvement, the patient was lost to follow-up.

## 3. Discussion

HZO can occur with or without ocular involvement in 10-20% of patients with a vesicular rash in the V1 dermatome [5]. Ocular findings most commonly include conjunctivitis, episcleritis/scleritis, keratitis, uveitis, and less commonly retinal necrosis which is often seen in immunocompromised patients [6].

VZV is considered a disease of late adulthood, with the rate of incidence increasing in each decade of life, especially when combined with immunocompromised individuals [7]. Although the majority of individuals affected by herpes zoster are over the age of 60 [8], recent trends show a decreasing mean age of onset which is likely due to widespread childhood varicella vaccination [9]. However, other risk factors have been documented for earlier age of onset including immunosuppression from either pharmacological or pathological etiologies (e.g., leukemia/lymphoma, HIV, transplant recipients, and corticosteroids or chemotherapy agents), autoimmune disorders, female gender, smoking, and diabetes [9].

In particular, smoking was the strongest risk factor for earlier age of onset, presenting on average 11.5 years earlier than nonsmokers [9]. Although approximately 30% of all individuals will develop at least one shingles outbreak in their lifetime, recurrent zoster infections are uncommon, ranging from 1.3% to 6.2% [10].

Our patient was a young, healthy 34-year-old individual who developed a recurrent HZO keratitis. He was not an active smoker, diabetic, or immunocompromised via the numerous etiologies listed above. Thus, it is possible that other factors may have contributed to his clinical presentation.

As an active weightlifter, our patient supplemented his diet with high-dose amino acids as well as anabolic steroids. We previously documented a case of recurrent HZO in a young, immunocompetent individual, preceded by high-dose L-arginine at 46.5 g/day [11]. L-arginine is a semiessential amino acid which has been found to have beneficial effects in wound healing, immune function, and metabolism at 4.2 to 20 g/day [12]. Nevertheless, the adverse effects of long-term supplementation above these recommended values have not been well studied [12].

L-Arginine also serves as an essential role in viral replication. Herpes simplex viruses (HSV-1 and HSV-2), varicella-zoster virus (VZV), cytomegalovirus (CMV), and adenovirus require arginine to replicate [13–16]. In vitro studies have demonstrated a decrease in HSV 1-2 replication, cell-to-cell transmission, and virus-mediated cytopathic effects in mediums deficient in L-arginine [13, 17]. Arginase, an enzyme that degrades L-arginine to L-ornithine and urea, was also found to be increased tenfold in a murine model of HSV-1-induced stromal keratitis infection [17], potentially linking arginine concentrations with symptom resolution. Other researchers also found an accumulation of arginine in the corneal epithelium during herpetic infections, a finding which was attributed to increased viral virulence and replication [17]. Additionally, the use of topical arginase led to a resolution of the herpetic episode, supporting arginine's role in infection and disease progression [18].

Despite these instances where arginine was associated with viral proliferation, other studies have shown that arginine and its derivatives can be viricidal. Naito et al. showed that arginine was effective against inhibiting HSV-1 replication in moderate concentrations while Yamasaki et al. reported that the arginine derivative N*α*-Cocoyl-L-arginine ethyl ester was effective at inhibiting HSV extracellular virus particles and replication [19, 20]. The contrasting results of these studies may be due to the variances of arginine being utilized. Isomers of arginine versus arginine complexes are likely to affect the structure and thus function of the amino acid. In most studies, supplementation of arginine did not exceed 30 g/day. Thus, there may be a critical threshold in which arginine dosage can lead to adverse effects which has not been clearly documented in the literature.

The other variable we believe may have influenced the recurrent episodes of HZO in our patient is his use of AAS. Anabolic steroids are not a commonly associated risk factor in HZV outbreaks. Nevertheless, these hormones have been documented to cause immunosuppressive effects. Grossman and Roselle found that one of the biological actions of androgen steroid hormones is the modulation of the immune system through the regulation of T lymphocyte function [2]. In particular, AAS with intact steroid nucleus exhibited a persistent immunosuppressive effect while those with nuclear alterations elicited a delayed immunostimulatory effect [2]. This distinction is important in this case, as our patient reported only taking AAS that had intact steroid nuclei.

The cells most adversely influenced by AAS with an intact steroid nucleus are lymphocytes (T cells) whose differentiation and proliferation are curtailed by reduced natural killer cytotoxic activity and the diminished production of certain cytokines such as IL-2, interferon-gamma (IFN-*γ*), and corticotropin [21]. These findings are supported in a separate study examining the impact of oxymetholone, another AAS with an intact steroid nucleus. Researchers found a 15% decrease in cytotoxic T cell activity measured in mice taking 300 mg/kg of oxymetholone for fourteen days, indicating that cell-mediated immunity was impaired following exposure [22]. Another study analyzed the role of testosterone in response to the influenza vaccine in animals. Furman et al. found that testosterone was immunosuppressive in vivo, downregulating transcription factors (such as FOS and JUN) implicated in immune activation [3]. One group of experiments demonstrated that orchiectomies of mice led to increased protection against viral, fungal, bacterial, and parasitic infestations [23]. Orchiectomized animals also rejected allografts more rapidly and had accelerated graft-versus-host reactions [23].

Lastly, the role of VZV reactivation within immunosuppressed patients has been analyzed. Patients with depressed cell-mediated immune function from hematopoietic stem cell transplantation were assessed for VZV reactivation while receiving intravenous *γ*-globulin antibodies. It was observed that the incidence of shingles markedly increased in the study patients, highlighting the importance of cell-mediated immune function in preventing viral reactivation [24].

Therefore, we hypothesize that AAS used by the patient may have compromised his cell-mediated immunity necessary to prevent VZV reactivation. Also, high-dose L-arginine supplementation may have also facilitated increased viral replication and virulence within a potentially immunocompromised individual. Nevertheless, we have only documented two similar cases and require further studies to deem if a real association exists.

## 4. Conclusion

In summary, we present a 34-year-old, healthy individual who developed recurrent HZO preceded by anabolic steroid abuse and high-dose L-arginine supplementation. In vitro experiments have demonstrated conflicting results between stimulatory versus inhibitory effects on herpes virus replication and virulence with L-arginine. Although data on arginine supplementation is limited, no long-term studies have documented the effects of high-dose arginine supplementation, greater than 20 grams a day, on immune function. Additionally, the immunosuppressive effects of AAS with intact steroid nucleus on the cell-mediated immune response have been documented in the literature. Increased rates of zoster reactivation have been witnessed in individuals who lack cell-mediated immune systems. Nevertheless, larger, prospective studies are needed to deem if a true association exists between recurrent HZO, L-arginine, and AAS.

## Figures and Tables

**Table 1:** The route, dosage, frequency, and schedule of anabolic-androgenic steroid (AAS) supplementation by the patient prior to each episode of HZO.

Anabolic androgenic steroid	Route	Amount	Frequency	Cycle
Trenbolone	Intramuscular	200 mg	1x week	4 weeks on 2 weeks off
Deca-durabolin	Intramuscular	200 mg	1x week	
Testosterone	Intramuscular	500 mg	1x week	

**Table 2:** A diet recall highlighting food and supplements containing high levels of L-arginine. The daily arginine intake ranged from 29.8–50.7 grams, averaging 40.2 grams/day. Arginine amount derived from the USDA National Nutrient Database (http://www.ars.usda.gov/nutrientdata).

Typical diet	Average amount	Arginine (grams)
Dry roasted nuts (peanuts, almonds, or walnuts)	2 cups	7.65-10.13
Meat (chicken, fish, pork, or beef)	4 cups	9.6-24.0
Whole cooked eggs	3 large	1.22
Cottage cheese	1 cup	2.56
Rice	2 cups	0.76
Supplements (protein powder, preworkout drinks, and amino acids)	Na^∗^	8.0-12.0
Patient range		29.8-50.7
Normal range		6.0-20.0
